# Use of TriClip steerable guide catheter for MitraClip: from bench to case

**DOI:** 10.1007/s12928-026-01296-w

**Published:** 2026-05-26

**Authors:** Fuk Kei Fong, Leo Kar Lok Lai, Kevin Ka Ho Kam, Alex Pui Wai Lee, Gilbert H. L. Tang, Kent Chak-yu So

**Affiliations:** 1 Division of Cardiology, Department of Medicine & Therapeutics, Prince of Wales Hospital, The Chinese University of Hong Kong, 30-32 Ngan Shing Street, Sha Tin, New Territories, Hong Kong SAR, China; 2 Li Ka Shing Institutes of Health Science, Prince of Wales Hospital, The Chinese University of Hong Kong, Hong Kong SAR, China; 3 Department of Cardiovascular Surgery, Mount Sinai Health System, New York, NY USA

A 77-year-old frail gentleman was referred for heart failure with severe atrial functional mitral (MR) and tricuspid regurgitation (TR) (Fig. 1 A and Videos 1–2). He underwent a combined MitraClip and TriClip transcatheter edge-to-edge repair (TEER) (Abbott) following heart team recommendation. The TriClip steerable guide catheter (SGC) was utilized for the combined TEER procedure, as described previously [1,2].

Despite multiple attempts, the transseptal puncture height was at 4.37 cm, slightly higher than planned largely attributable to a markedly enlarged left atrium secondary to atrial fibrillation (Fig. 1B). During MitraClip TEER, the clip delivery system (CDS) necessitated deep advancement, which compromised its guide tip control and leaflet grasping (Figs. 1 C-D; Video 3). To mitigate this, we utilized the L-knob of the TriClip SGC to reduce the height from mitral valve, thereby enhancing the guide tip manoeuvrability and steering accuracy (Fig. 1 E; Video 4). This adjustment facilitated the successful deployment of MitraClip in an optimal position, markedly reducing MR from severe to mild-moderate (Fig. 1 F; Videos 5–6). Tricuspid TEER was then successfully performed, decreasing TR severity from severe to mild. The patient was subsequently discharged 2 days later. Three-month echocardiogram at follow-up showed a sustained reduction in MR severity to mild to moderate (Videos 7–8).1 .

A bench test was conducted, demonstrating that the shorter, 1.5 cm curved tip of TriClip SGC—compared to the standard MitraClip SGC with 2.5 cm tip (Fig. 1 G)—will confer to a 5 mm taller height from the mitral valve to its guide tip (Figs. 1 H-I). This suggests that a lower transseptal height is needed for Mitral TEER when using off-label TriClip SGC compared to the standard MitraClip SGC. Besides, the S/L knob on the TriClip SGC allows for further height adjustments— L knob to shed height (Figs. 1 J & L; Video 9) and S knob to gain height (Figs. 1 M & O; Video 10), accommodating for suboptimal transseptal positions. When we apply the L knob, there will be a secondary medial dive trajectory which can be corrected by reducing the M knob as demonstrated in the bench test (Fig. 1 K); vice versa, when we manipulate the S knob to gain substantial height, there will be a secondary lateral dive trajectory which can be corrected by increasing the M knob back (Fig. 1 N). This is different from using the MitraClip SGC for M-TEER. In the MitraClip SGC, we can torque the guide posteriorly to gain height, which often requires the addition of the “anterior” and “M” knobs in order to compensate for the associated secondary movements.

We believe that the integration of a single TriClip SGC not only facilitates combined mitral and tricuspid TEER procedures, but may also be advantageous during M-TEER when transseptal puncture compromise is anticipated, such as in patients with small atria (e.g., in acute MR or specific Asian anatomies) or large atria due to atrial secondary pathologies. Further bench testing and clinical studies are essential to systematically evaluate the functionality of the S/L knob, their secondary motion and limits, and assess the broader applicability of the TriClip SGC in mitral TEER procedures.

**Fig. 1:**
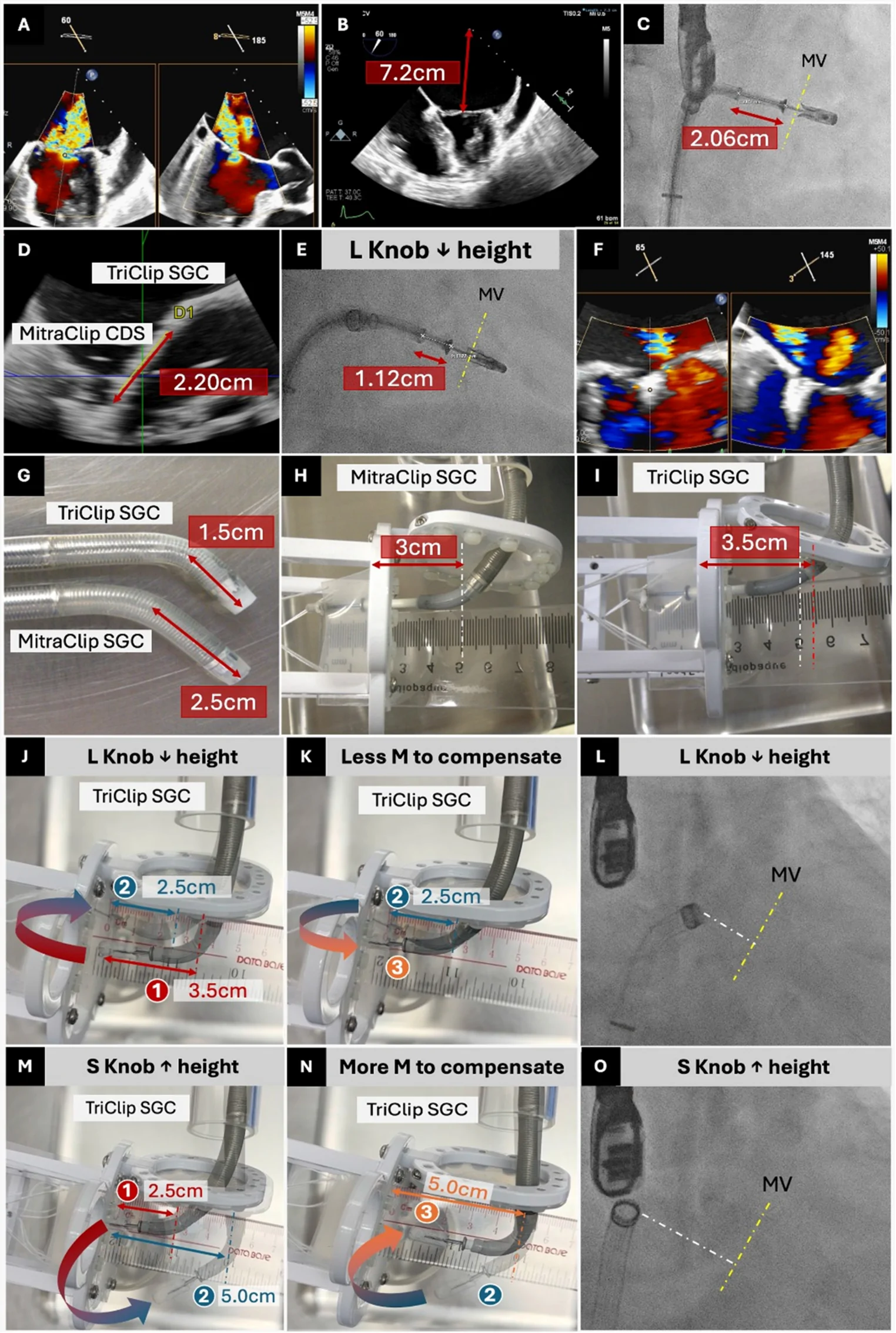
**A**-**B**: Severe mitral regurgitation (MR) originating from the A2-P2 scallops and a severely dilated left atrium secondary to atrial fibrillation. Figures **C**-**D**: The MitraClip delivery system (CDS) had to be deeply advanced, compromising its guide tip control and alignment after a suboptimal transseptal height. Figure **E**: The height has been reduced in relation to the mitral annulus after manipulating the L-knob on TriClip SGC, thereby enhancing its guide tip maneuverability and steering accuracy of MitraClip. Figure **F**: Mild-moderate residual MR after MitraClip. Figure **G**: Bench test comparing the shorter 1.5 cm curved tip of TriClip SGC and the standard MitraClip SGC with a 2.5 cm tip. Figures **H**-**I**: The TriClip SGC with a shorter curved tip therefore reduces the transseptal height requirement by 5 mm for left-sided MitraClip procedure (as off-label use). Figures **J**-**L**: By adding the L-knob on the TriClip SGC, we can shed height (e.g. from 3.5 cm to 2.5 cm) in cases of suboptimal transseptal puncture. This is accompanied by a secondary medial dive trajectory which can be corrected by reducing the M knob. Figures **M**-**O**: By adding the S-knob on the TriClip SGC, we can gain height (e.g. from 2.5 cm to 5 cm) in cases of suboptimal transseptal puncture. A secondary lateral dive trajectory is observed which can be corrected by increasing the M knob

## Supplementary Information

Below is the link to the electronic supplementary material.

Supplementary Material 1

Supplementary Material 2

Supplementary Material 3

Supplementary Material 4

Supplementary Material 5

Supplementary Material 6

Supplementary Material 7

Supplementary Material 8

Supplementary Material 9

Supplementary Material 10
